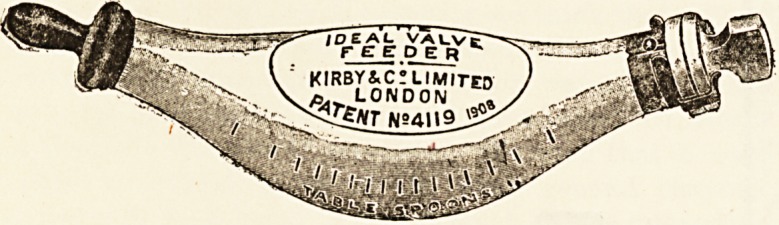# New Appliances and Things Medical

**Published:** 1908-11-21

**Authors:** 


### NEW APPLIANCES AND THINGS MEDICAL.

[We shall be glad to receive at our Office, 28 & 29 Southampton Street, Strand, London, W.C., from the manufacturers, specimens of all new preparations and appliances which may be brought out from time to time.]

### AN IMPROVED FEEDING BOTTLE.

We have received from Messrs. H. and T. Kirby and Co., Limited, of 14 Newman Street, London, a specimen of their "Ideal Valve" Infant Feeder. The special point of this feeding bottle consists, as its name implies, in the construction and arrangement of the inlet air-valve. This reduces the risks of contamination of the milk to a minimum since no indiarubber enters into its composition, and it can be sterilised with ease. The valve-stopper is of solid glass, which is ground to fit accurately into the bottle neck, and a simple metal-clip attachment prevents the stopper falling out or leaking and aids in regulating the air entry. The bottle itself is boat-shaped, and possesses all the advantages of the best modern feeding bottles. It is open at both ends and can be flushed thoroughly under a stream of water; it has no corners for the lodgement of decomposing matter, and it is graduated in tablespoons and ounces. We have tried this feeder, and find that the valve is easily applied according to the directions given, and that it is efficient. It can be arranged without difficulty to permit the entrance of an even supply of air behind the column of milk, and this can be regulated according to the necessities of the case. The metal clamp is durable, and can be taken off and reapplied without difficulty. The feeder is fitted with a non-collapsible glazed teat of transparent indiarubber, which is not injured by boiling water. The specimen sent to us has a single round hole, but a leech-bite opening can also be supplied. The "Ideal Valve" Feeder is sold at the price of 1s. 6d. complete, or post free for 1s. 10d., and spare parts can be obtained at a proportionate rate. Medical men will be supplied with samples free of cost on application to the manufacturers at the above address. The feeder certainly seems to meet a distinct want, and should be welcomed by medical practitioners and their patients.

### RIGOLLOT'S MUSTARD PAPER.

Samples have been forwarded to us of Rigollot's Mustard Papers, which are almost as well known in this country as they are among the medical practitioners of France. These papers have various merits. They are very clean and can be applied with the least possible expenditure of time and trouble. In the neat tin cases in which they are supplied, they can be carried about in the pocket or kept securely in any climate without risk of deterioration. They can be used wherever a mustard leaf or mustard plaster is indicated, and from actual experience we know them to be rapid and efficient in their action. Rigollot's papers are sold in three different shapes. They are manufactured in Paris, but there is an English depôt at 67 Southwark Bridge Road, London, S.E. (They are still used in the principal hospitals of Paris and in the French military hospitals, and they have also been supplied to the navies of Great Britain and of France.)

### HOSPITAL SANITARY ARRANGEMENTS.

The water-closets used throughout the building of the Manchester Royal Infirmary were supplied by Messrs. Doulton and Company, Limited, and are of special design. They are of the wash-down type, made in strong white-glazed Vulcan-ware, with extension for building into wall, the closet being quite clear of the floor, and cistern of the same material is fixed just above the pan and is fitted with a large float-valve which is actuated by the door. The cistern is so arranged that the closet is only flushed when the door is opened as the user leaves; when entering, the tank does not discharge. The usual door-action arrangement means a double discharge, and accordingly a great waste of water. The baths are fitted with Doulton's patent mixing valves, which regulate the temperature by a slight movement of a lever, and the same pattern valve is fixed over the hospital lavatories. These are made with a lengthened lever that can be moved by the elbow.

## Figures and Tables

**Figure f1:**